# A Complex Case of Bilateral Carpal Tunnel Syndrome in a Patient With Gout: A Case Report and Review of Literature

**DOI:** 10.7759/cureus.70493

**Published:** 2024-09-30

**Authors:** Lyubomir Gaydarski, Kristina Petrova, Asen Hadzhiyanev, Boycho Landzhov, Georgi P Georgiev

**Affiliations:** 1 Department of Anatomy, Histology and Embryology, Medical University of Sofia, Sofia, BGR; 2 Department of Clinical Laboratory, Medical University of Sofia, Sofia, BGR; 3 Department of Neurological Surgery, University Hospital "St. Rilski", Sofia, BGR; 4 Department of Orthopaedics and Traumatology, University Hospital Queen Giovanna - ISUL, Sofia, BGR

**Keywords:** accessory muscle, carpal tunnel syndrome, diagnostics, gout, surgery

## Abstract

Carpal tunnel syndrome (CTS) is the most common nerve entrapment condition of the upper extremity, primarily caused by compression of the median nerve as it passes through the carpal tunnel in the wrist. While various factors can lead to this condition, the accumulation of gouty tophi in the flexor tendons is a rare cause. This case involves a 57-year-old manual laborer who presented with progressively worsening symptoms in both hands, including numbness, pain, and muscle weakness, which were more severe in his right hand. He had a 20-year history of gout, managed with medication. On examination, significant findings included atrophy of the thenar muscles, gouty tophi in both hands and a palpable soft tissue mass in the right wrist. Diagnostic tests confirmed CTS in both hands, with more pronounced severity on the right side. Imaging studies revealed the presence of gouty tophi and soft tissue masses in both hands. Surgical decompression of the carpal tunnel was performed on each hand, with excision of the soft tissue mass in the right wrist, which was identified as synovitis of the flexor tendons. During surgery, an accessory flexor muscle of the fifth finger was discovered in the right hand. Postoperative recovery was smooth, and the patient showed significant improvement in symptoms at follow-up. This case underscores the complex and multifactorial nature of CTS, especially when associated with gout. The combination of gout and repetitive physical strain from the patient’s occupation likely contributed to the development of the condition in his right hand. Although the accessory flexor muscle was not the primary cause of nerve compression, its presence highlights the importance of considering anatomical variations when diagnosing and managing CTS. This case enhances the current understanding of CTS and emphasizes the need for personalized diagnostic and treatment strategies in patients with underlying conditions such as gout.

## Introduction

Carpal tunnel syndrome (CTS) is the most commonly diagnosed entrapment neuropathy of the upper extremity, caused by compression of the median nerve (MN) as it passes through the carpal tunnel in the wrist [1,2]. CTS can develop due to various factors, including space-occupying lesions, hand trauma leading to hemorrhage, repetitive hand or wrist movements, and anatomical variations such as a smaller carpal tunnel, abnormal muscles or tendons, and a persistent median artery [2]. Medical conditions like diabetes, rheumatoid arthritis, tophaceous gout, obesity, and hypothyroidism also contribute to CTS [2]. Although rare, gouty tophi accumulating in the flexor tendons can cause both chronic and acute CTS, with an estimated incidence of 0.6% [3]. Common symptoms include numbness, pain, paresthesia, and muscle weakness, with patients with gout often experiencing more severe and persistent CTS symptoms [4]. Diagnostic methods include physical examinations that assess Tinel's sign, Phalen's test, and the carpal compression test [5], along with assessments using the Arm, Shoulder, and Hand (DASH) score, the Boston Carpal Tunnel Questionnaire (BCTQ), and the Pittsburgh Sleep Quality Index (PSQI) [6]. Imaging techniques like ultrasound (US), computed tomography (CT), and magnetic resonance imaging (MRI) are crucial in diagnosing CTS [7-9]. Treatment options range from anti-inflammatory drugs and physical therapy to corticosteroids (administered orally or via local injection), bracing, and surgical decompression [10]. This report discusses the case of a 57-year-old male with bilateral CTS, tophaceous gout, and an accessory flexor of the fifth finger in the right hand, along with a brief review of the literature on the diagnosis, imaging, pathological findings, and management of CTS due to gouty tophi.

## Case presentation

A 57-year-old male manual worker was referred to our department for surgical management of carpal CTS. He presented with typical symptoms in both hands, including paresthesia, nocturnal pain, stiffness, intermittent numbness in the thumb, index, and middle fingers, and difficulty gripping objects. The symptoms were more severe in his dominant right hand, where a preoperative soft tissue mass was also observed proximal to the carpal tunnel. His symptoms began 10 years ago, initially affecting the right hand and later involving the left. The patient delayed seeking treatment, but over time, the symptoms progressively worsened, with wrist extension exacerbating his discomfort. He reported undergoing multiple courses of conservative treatment, including anti-inflammatory drugs, physiotherapy, and nighttime wrist orthoses, but these measures provided only limited relief. The patient also had a 20-year history of gout, with three to four attacks per year, initially managed with Milurit (allopurinol) 100 mg daily and later with Adenuric (febuxostat) 120 mg daily, along with dietary adjustments. During his treatment for gout, as well as before the surgeries, his uric acid levels remained within normal ranges (Table 1).

**Table 1:** Laboratory results ESR: erythrocyte sedimentation rate; WBC: white blood cells; LYM: lymphocytes; MO: monocytes; GRAN: granulocytes; RBC: red blood cells; HGB: hemoglobin; HCT: hematocrit; MCV: mean corpuscular volume; MCH: mean corpuscular hemoglobin; MCHC: mean corpuscular hemoglobin concentration; RDW: red cell distribution width; PLT: platelets; CRP: C-reactive protein; AST: aspartate aminotransferase; ALT: alanine aminotransferase; INR: international normalized ratio

Parameters (units)	Results	Reference ranges (male)
ESR (mm/h)	23	<50 years up 20, >50 years up 30
WBC (10^9^/L)	6.37	3.5-10.5
LYM (10^9^/L)	1.2	0.6-4.1
LYM (%)	19.7	20-40
MO (10^9^/L)	0.71	0.20-1.50
MO (%)	11.1	3-13
GRAN (10^9^/L)	3.71	2-7.8
GRAN (%)	58.21	44-76
RBC (10^12^/L)	4.16	4.2-6.2
HGB (g/L)	125	140-180
HCT (L/L)	0.42	0.37-0.55
MCV (fL)	95	82-98
MCH (pg)	32	26.5-32
MCHC (g/L)	337.4	295-360
RDW (%)	12.2	11.5-14.5
PLT (10^9^/L)	197.4	130-440
Chloride (mmol/L)	105	98-107
Sodium (mmol/L)	139	135-145
Potassium (mmol/L)	5.2	3.5-5.1
Glucose (mmol/L)	4.97	3.3-6
Urea (mmol/L)	15.9	1.7-8.30
Creatinine (µmol/L)	174	50-133
CRP (mg/dL)	5.66	0-0.6
Uric acid (µmol/L)	348	214-458
Protein total (g/L)	82	63-84
Albumin (g/L)	44	35-50
AST (U/L)	57	0-41
ALT (U/L)	99	0-42
INR (INR)	1	0.85-1.25

**Figure 1:**
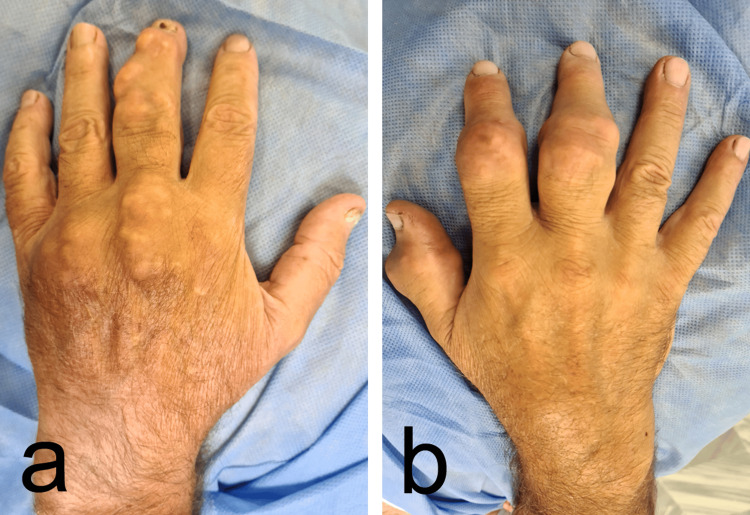
Photograph of the patient's hands showing the presence of gouty tophi (a) Left hand; (b) right hand

He denied any family history of gout or CTS. On physical examination, there was atrophy of the thenar muscles in both hands, which was more pronounced on the right. Gouty tophi were noted, particularly in the interphalangeal joint of the thumb, the proximal interphalangeal joints of the second and third fingers on the right hand, the metacarpophalangeal joints of the third and fourth fingers on the left hand, and the proximal and distal interphalangeal joints of the third finger on the left hand (Figures 1a, 1b).

**Figure 2:**
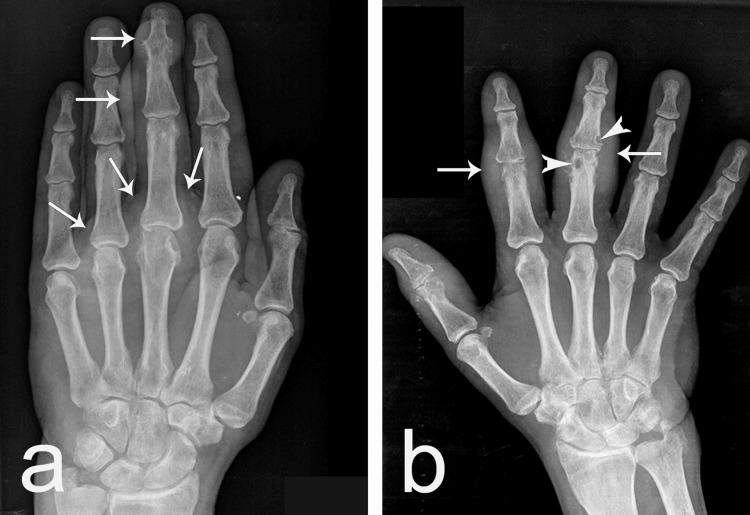
X-rays of both hands of the patient (a) Left hand; (b) right hand; white arrows: gouty tophi presented as soft tissue mass; white arrowheads: bone cysts

X-rays of the left hand showed tophi as soft tissue masses, along with sclerotic borders, irregular spicules on the middle phalanx, and ankylosis of the distal interphalangeal joint. On the right hand, X-rays revealed sclerotic borders and irregular spicules on the proximal phalanx, as well as a lytic lesion. Another lytic lesion was detected in the middle phalanx of the same hand. Tophi were also observed in the affected areas of the hand (Figure 2).

**Figure 3:**
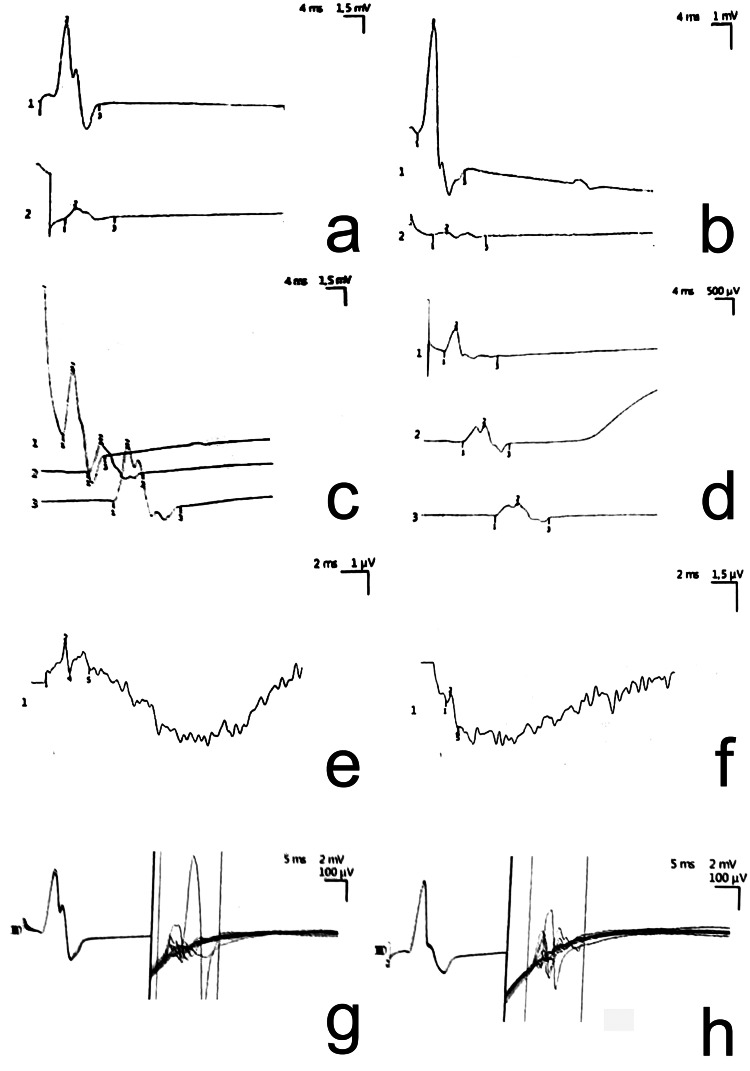
EMG graphical results (a) Right abductor pollicis brevis, through canalis carpi, n. medianus, C8-T1; (b) Left abductor pollicis brevis, through canalis carpi, n. medianus, C8-T1; (c) Right abductor pollicis brevis, n. medianus, C8-T1; (d) Left abductor pollicis brevis, n. medianus, C8-T1; (e) Sensory conduction speed of the right n. medianus, C8-T1; (f) Sensory conduction speed of the left n. medianus, C8-T1; (g) F-wave of the right abductor pollicis brevis, n. medianus, C8-T1; (h) F-wave of the left abductor pollicis brevis, n. medianus, C8-T1

Tinel's sign, Phalen's test, and the carpal compression test were positive in both hands, with more pronounced results on the right. The DASH score for the right hand was 58, the BCTQ averaged 3-4 across sections, and the PSQI was 10. For the left hand, the DASH score was 52, the BCTQ averaged 2-3 across sections, and the PSQI was 6. Electromyography (EMG) confirmed reduced conduction and sensory function of the MN in both hands, slightly worse on the right (Figure 3).

**Figure 4:**
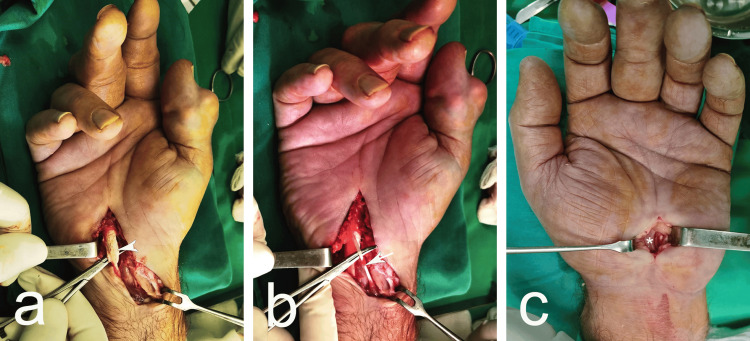
Intraoperative photographs of the right hand (a and b) and left hand (c) White arrowhead: tendon of the m. flexor digitorum superficialis, infilled with gouty tophi; white arrow: tendon of the accessory flexor muscle of the fifth finger; asterisk (*): median nerve

Laboratory results are summarized in Table 1. Surgical intervention was planned, starting with the right hand, due to the worse clinical manifestation, followed by the left hand six months later. The procedures were performed under local anesthesia with a tourniquet. The patient was positioned supine, with the hand, wrist, and fingers slightly extended, supported by a small pillow under the wrist. On the right hand, a classical surgical approach for CTS was used, extending the incision proximally in a zigzag pattern to excise the soft tissue mass, which was found to be synovitis of the flexor tendons (Figure 4a). On the left hand, a longitudinal incision approximately 1.5-2 cm long was made along a vertical line extending from the third interdigital space, ending 0.5 cm proximal to the distal wrist crease (Figure 4c). In both hands, after the skin incision, the surgery proceeded classically. The underlying tissues were carefully incised through the palmar aponeurosis until the flexor retinaculum (FR) was exposed. Using a mosquito clamp, the FR was incised from the distal to the proximal end along its ulnar side. The distal edge of the FR and the branch of the MN were then exposed. Additionally, the distal fibers of the volar carpal ligament were severed after elevating the skin at the proximal end of the wound. An accessory superficial flexor muscle to the fifth finger was also observed in the right hand (Figure 4b).

In both hands, the carpal canal was inspected visually and manually before the skin incision was sutured closed. After surgery, the hands were immobilized with plaster splints for 14 days. On the 14th day, the stitches were removed, and the patient began a 10-day course of physiotherapy. At the six-month follow-up, the patient showed significant improvement in his carpal tunnel scores. For the right hand, the DASH score was 21, the BCTQ ranged between 1 and 2 across categories, and the PSQI was 4. For the left hand, the DASH score was 18, the BCTQ averaged 1 across categories, and the PSQI was 3. Two years of routine follow-up revealed no signs of CTS recurrence in either hand.

## Discussion

CTS caused by gouty tophi is a rare condition, with only a few documented cases in the literature [3]. The rarity of gouty related-CTS (GCTS) stems from the fact that tophi is an uncommon cause of CTS [3]. However, the increasing prevalence of gout, likely due to dietary changes, has led to greater awareness of its complications, including GCTS. This heightened awareness underscores the importance of a thorough medical history, physical examinations, and appropriate imaging studies to prevent misdiagnosis and missed cases [11].

Several case studies illustrate the variability and complexity of GCTS presentations. For instance, Luo and Zhang described a case where GCTS manifested subtly in a young patient whose gout was only diagnosed after CTS symptoms worsened, revealing large tophi compressing the MN [12]. This case highlights the importance of considering gout in differential diagnoses, even in younger patients without a prior gout diagnosis. Similarly, Buruian et al. showed that severe, chronic gout can lead to significant intratendinous and intramuscular tophi, directly compressing the MN and complicating the clinical picture [13]. Hao et al. reported a case where gouty tophi was found in a patient without a known history of gout but with elevated uric acid levels, suggesting that hyperuricemia alone might be a warning sign for potential GCTS [14]. Zhang et al. provided evidence that untreated, long-standing gout can result in extensive tophi deposition, including beneath the epineurium of the MN, causing bilateral neurological deficits [15]. Yan et al. supported this by showing that long-term untreated gout can lead to substantial compression of the flexor tendons by urate crystals, detectable through advanced imaging techniques [16]. Finally, Calderon and Chung highlighted that asymptomatic hyperuricemia could lead to postoperative complications, with gout potentially first manifesting or becoming clinically significant after surgical intervention [17]. These cases collectively enhance our understanding of GCTS, showcasing its diverse presentations and emphasizing the critical need for thorough evaluation of at-risk patients. The cases discussed are summarized in Table 2.

**Table 2:** Summary of gout-related carpal tunnel syndrome cases

Authors	Age	Sex	Preoperative uric acid level (mmol/L)	Location	Symptoms	Treatment	Postoperative complications
Calderon MS, Chung KC, 1999 [17]	69	Male	0.47 (0.25-0.47 for males over 60 years)	Right hand	Dysaesthesia and decreased grip strength	Surgery	First: Postoperative scar band over the median nerve; second: Erythematous incision.
Yan GE et al., 2016 [16]	54	Male	0.61 (0.27-0.48 for males under 60 years)	Bilaterally, more severe in the right hand	Palmar numbness and disability	Conservative	-
Luo PB, Zhang CQ, 2018 [13]	25	Male	0.57 (0.27-0.48 for males under 60 years)	Right hand	Numbness in the fingers and decreased grip strength	Surgery	Uneventful 12 months follow-up.
Buruian A et al., 2022 [12]	52	Male	0.24 (0.27-0.48 for males under 60 years)	Left hand	Wrist pain, edema, and numbness	Surgery	Uneventful 18 months follow-up with mild pruritus over the scar.
Zhang W et al., 2023 [15]	64	Male	0.60 (0.25-0.47 for males over 60 years)	Left hand	Numbness and tingling in the fingers	Surgery	Uneventful 12 months follow-up.
Hao H et al., 2023 [14]	37	Male	0.49 (0.27-0.48 for males under 60 years)	Right hand	Numbness in the fingers	Surgery	Uneventful 12 months follow-up.
Present case	57	Male	0.35 (0.27-0.48 for males under 60 years)	Bilaterally, more severe in the right hand	Paresthesia, pain, stiffness, numbness, decreased grip strength	Surgery	Uneventful 24 months follow-up.

In our case, it is noteworthy that tophi deposits were observed in the flexor tendons of the right dominant hand, along with an accessory flexor muscle for the fifth finger. It can be speculated that these factors, particularly the impaired tendons, contributed to the development of CTS in the right hand by narrowing the carpal tunnel space and worsening symptoms compared to the left hand. We believe that the thin tendon of the variant fifth superficial flexor, with its delicate morphology, likely did not significantly contribute to this condition. However, this raises the question of how gout could be a provoking factor for CTS in the contralateral hand, where there were no tendon deposits and uric acid levels were within normal ranges. Therefore, we consider this case an excellent example of the complex etiology of CTS.

Neurological symptoms such as pain, paresthesia, and numbness along the distribution of the MN are typical manifestations of CTS [4]. These sensory symptoms are crucial not only for diagnosing CTS but also for evaluating the effectiveness of treatment [18]. Among the diagnostic tools, Tinel's sign and Phalen's maneuver are commonly used. Tinel's sign is considered positive when tapping on the MN at the wrist triggers symptoms, while Phalen's maneuver is positive if symptoms occur when the wrist is held in forced flexion for one minute [19]. Although these tests are easy to perform, their sensitivity and specificity are widely debated. Phalen's maneuver has a reported sensitivity ranging from 42% to 85%, while Tinel's test shows a broader sensitivity range of 38% to 100%. The specificity for Phalen's maneuver varies from 54% to 98%, and for Tinel's test, it ranges from 55% to 100% [19]. In addition to these physical tests, various questionnaires, such as the DASH score, BCTQ, and PSQI, are used to assess the severity of CTS [6]. The BCTQ is particularly noteworthy as it includes both a Symptom Severity Scale (SSS) and a Functional Status Scale (FSS) [20]. Recently, the BCTQ was adjusted for our country, where it has proven to be a reliable and effective tool for assessing patients with CTS both before and after surgery [20]. The EMG often reveals decreased MN conduction velocity and impaired sensory function in CTS patients [13-15]. Imaging diagnostics are also essential, with X-rays detecting bone lesions, erosions, sclerotic borders of bones, and irregular spicules, while US, CT, and MRI provide further evaluation. The US is favored for its high resolution and cost-effectiveness, offers precise visualization of wrist structures, and can distinguish between intra- and extraneural masses [7,8]. Therimadasamy et al. noted that the US is particularly effective in differentiating gouty tophi from nerve tumors [8]. Chen et al. found MRI superior for defining the extent and location of tophi, while CT was more effective in detecting calcifications within tophi, though these methods are less cost-effective than X-rays and US [9].

Treatment for CTS can be either conservative or surgical. Conservative treatments include physiotherapy, corticosteroid administration, and bracing to relieve symptoms. For patients with GCTS, managing uric acid levels is also essential [10,14,15]. However, these treatments do not eliminate tophi or decompress the MN, so surgery is often necessary to prevent permanent nerve damage. Surgical intervention to decompress the nerve and remove tophi is recommended to prevent complications like joint deformities and tenosynovitis [15,16]. Although wound healing can be challenging due to the inflammatory nature of gout, our case did not present any such issues. However, residual tophi may persist, leading to disease recurrence [14]. Postoperative preventative measures, including uric acid-lowering drugs and dietary modifications, are crucial to avoid future gout attacks [14]. The primary surgical goal in CTS is to cut the FR to decompress the MN. The traditional open surgical approach, while providing excellent visualization of anatomical variations, is sometimes debated due to complications like local pain, hypersensitivity, and scar tissue formation [6,21]. Endoscopic and mini-incision techniques have been developed to minimize these complications. Mini-incision techniques offer advantages such as smaller scars and faster recovery, but they also have limitations, including reduced visibility and the potential for inadequate decompression [6,20,22]. In the reported case, a classical approach was preferred on the right side due to the need for synovectomy of the tendons proximal to the carpal tunnel. On the left side, a mini-incision approach, as described by Georgiev and Karabinov [6], was performed.

During our patient's surgery, an accessory superficial flexor muscle for the fifth finger was discovered in the right hand. However, due to the limited surgical incision, we were unable to determine the muscle's origin or insertion points precisely. As Georgiev et al. highlighted, a limited skin incision during decompression surgery may not fully reveal all anatomical variations. Therefore, it is crucial to address any variant anatomical structures encountered during surgery, ideally without extensive dissection [23]. This muscle variant is similar to one reported by Bale and Herrin, though in their case, it was present bilaterally, whereas our case was unilateral [24]. Accessory muscles are commonly found in the upper limbs, but their concurrent presence with CTS is rarely documented, occurring in only 13% of operated cases [1]. Börekci et al. noted that most accessory muscles are identified during surgery, with few preoperative diagnoses using US or MRI [1]. In cases where accessory muscles do not compress the MN, open surgery with careful preservation of these muscles is recommended [1]. It is important to note that the presence of an accessory muscle does not necessarily result in compression neuropathy [25].

## Conclusions

The present case report highlights the complex and multifactorial nature of CTS, especially when associated with gouty tophi. Our findings emphasize the presence of gouty tophi around the tendons and muscles in the carpal tunnel only in the patient's dominant right hand. CTS was diagnosed and surgically managed in the left hand as well, yet no manifestation of gouty tophi was found. We hypothesize that the combination of physical strain from the patient's occupation and underlying gout likely contributed to the CTS in the right hand. Additionally, the discovery of an accessory flexor muscle for the fifth finger in the right hand is particularly noteworthy. While we believe this variant muscle did not significantly contribute to the compression of the MN, its presence illustrates the anatomical variability that can complicate CTS. This case underscores the importance of considering both gout and anatomical variations when diagnosing and treating CTS, particularly in patients with asymmetrical symptoms. Therefore, we believe this case enriches the current literature on CTS and will be beneficial for medical specialists across various fields.
